# The Importance of the Entry Point and Osteotomy Direction in Calcaneal Lengthening Osteotomy

**DOI:** 10.1177/24730114251363916

**Published:** 2025-09-01

**Authors:** Pascal Raffael Furrer, Arnaud Klopfenstein, Silvan Beeler, Arnd Fredrik Viehöfer, Stephan Hermann Wirth

**Affiliations:** 1 Department of Orthopaedics, Balgrist Hospital, University of Zurich, Switzerland

**Keywords:** calcaneal lengthening osteotomy, Hintermann osteotomy, intraarticular osteotomy, safe zone for osteotomy, Ideal osteotomy, flat foot correction

## Abstract

**Background:**

The Hintermann osteotomy (HOT) is one type of calcaneal lengthening osteotomy during progressive collapsing foot deformity surgery. The entry point on the lateral wall of the calcaneus is critical because it affects the direction and depth of the osteotomy. Accurate osteotomy placement can be technically demanding, and joint facets can sustain damage in up to 50% of the cases. We hypothesize that the further posterior the osteotomy is performed, the greater the risk of facet injury.

**Methods:**

Twenty-two computed tomography–based 3-D models underwent simulated HOT at 5, 10, 15, 20 mm posterior to the calcaneocuboid joint. Primary outcomes were facet penetration rate and “safe-zone” angle; secondary outcomes were distance to the flexor hallucis longus (FHL) and anterior-facet translation.

**Results:**

Facet penetration increased from 0% (0/22) with entry points 5 to 15 mm posterior to the calcaneocuboid joint to 23% (5/22) at 20 mm. The safe-zone angle narrowed from 11 ± 2.6 degrees at 5 mm to 3.0 ± 6.5 degrees at 20 mm (*P* < .01). Mean FHL clearance decreased from 44 ± 6 mm to 35 ± 6 mm (−20%, *P* < .05), and anterior-facet translation increased by 32% between the 5- and 20-mm cuts.

**Conclusion:**

The choice of the entry point is crucial. If an entry point is chosen 20 mm behind the calcaneocuboid joint, facet penetration is anatomically inevitable in 23% of cases. A more anterior entry point results in a longer distance between the lateral wall and the sensitive medial structures.

**Level of Evidence:**

Level IV, case series.

## Introduction

Operative correction of progressive collapsing foot deformity often requires a number of sequential steps involving a combination of soft tissue and osseous surgical interventions.^1^ A lateral column lengthening osteotomy of the calcaneus is usually performed to address increased forefoot abduction and improve the talonavicular coverage.^2-6^ The Hintermann osteotomy (HOT) is an incomplete opening wedge osteotomy and is performed between the middle and posterior facet of the calcaneus.^2,3,7^ Because of the anatomical position of the facets and limited intraoperative overview, it is often difficult to determine the correct osteotomy plane; thus, the anatomy of the subtalar joint varies by individual and population.^8-10^ Incorrect osteotomy is described in more than 50% of cadaveric specimens, can injure the cartilage of the subtalar joint facets, the sustentaculum tali, the neurovascular and tendon structures in the tarsal tunnel, or provoke subtalar instability due to lesions to the interosseous structures.^11-14^ Because joint-preserving surgery in progressive collapsing foot deformity is performed in patients with healthy subtalar joint condition, there is a significant risk of premature joint degeneration in the event of facet penetration.^6,10,11^ The osteotomy plane is described in the original description of the HOT with an arbitrary entry point of about 15 mm (12-20 mm) behind the calcaneocuboid (CC) joint and a direction parallel to the posterior facet, perpendicular to the ground.^2,4^ Individual arbitrary entry points were also chosen in follow-up investigations to determine the optimal osteotomies.^15^ The angle to be osteotomized varies depending on the chosen entry point, and so it may also change the range of osteotomies that can be performed without penetration of the facets. In addition, depending on the entry point, the exact osteotomy depth must be defined to prevent medial wall transection and thus protect the structures in the tarsal tunnel and the sustentaculum tali. Furthermore, by opening the incomplete osteotomy, the displacement of the osteotomized anterior fragment varies according to the entry point.

The purpose of this study was to demonstrate the effect of different entry points, the safest osteotomy level to avoid damaging any joint facet, the depth of the ideal cut that does not violate the medial structures, and the effect of the different osteotomy planes on the transposition of the anterior fragment.

## Materials and Methods

### Patient Collection

Previously segmented computed tomographic (CT) scan data were collected retrospectively and used for 3-dimensional modeling and calculation. Included were all consecutive CT scans that had already been segmented to serve as a template for a corrective osteotomy of the contralateral foot. All CT examinations were carried out in the supine position (nonweightbearing) with a slice thickness of 1 mm (120 kV, Philips Brilliance 40CT; Philips Healthcare, the Netherlands), and images were segmented using Mimics software (Materialise, Leuven, Belgium). 3D analysis was carried out with in-house developed software CASPA (Balgrist University Hospital). Patients with documented pathology or complaints on the side to be examined were excluded from participating. Informed patient consent and approval from the local ethical committee (BASEC-Nr. Req-2023-01591) were obtained.

### Anatomical Analysis

**Figure 1.**
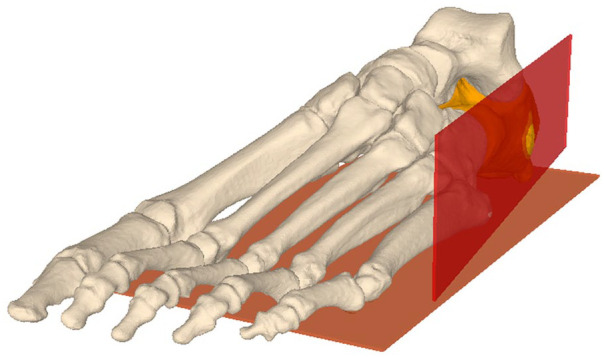
The plane of the planta pedis and the lateral wall are illustrated. The osteotomies were simulated at a perpendicular angle to the planta pedis.

**Figure 2.**
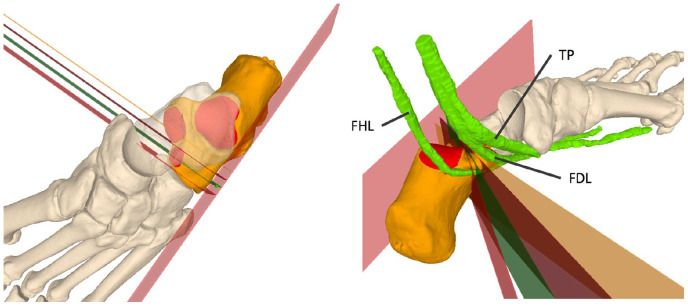
Left: The lateral wall with its predefined entry points (5, 10, 15, and 20 mm posterior to the CC joint) is shown. These are demonstrated perpendicular to the lateral wall, which was defined as 0 degrees in our analysis. Right: Osteotomies in different directions for the HOT between the posterior and middle facet, depending on the entry point and its relation to the medial structures. The 3 tendons of the flexor hallucis longus (FHL), tibialis posterior (TP), and flexor digitorum longus (FDL) muscles are shown in green. HOT, Hintermann osteotomy.

The osteotomies and anatomical analyses were performed in a predefined manner. The feet were aligned using the talus dome for coronal alignment and the lowest part of the calcaneus and the fifth metatarsal for flexion and extension so that the osteotomy could be performed perpendicular to the planta pedis, as shown in Hintermann’s surgical description. In the “best-fit” procedure, a plane was then placed on the lateral wall to obtain a reference of the osteotomy angle (Figure 1). The calcaneal articular surface of the facet were marked manually on the segmented calcaneus as previously published.^16^ The FHL tendon was located and marked manually on each slice of the scan. The osteotomies were then simulated perpendicular to the planta pedis. Different osteotomies were simulated with lateral wall entry points 20 mm, 15 mm, 10 mm, and 5 mm posterior to the CC joint, which corresponds to a gradual and newly selected interval (Figure 2).

**Figure 3.**
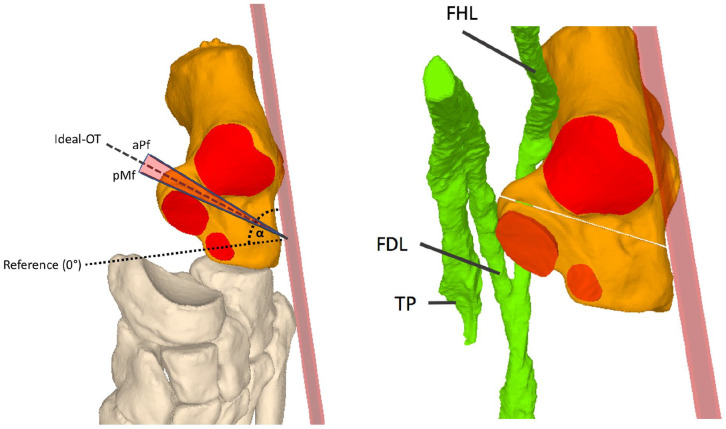
Left: Illustration of 2 osteotomies that were simulated and calculated to make precise contact with the facets but not penetrate them (pMf, aPf). The range in between was called the “safe zone,” where an osteotomy would not penetrate the joint facet. The ideal osteotomy is the bisection between the 2 facets indicating the safest possible osteotomy. The reference line is the perpendicular to the lateral wall, which was set at 0 degrees. Osteotomies performed anterior towards the CC joint were marked with a positive sign, whereas those performed posteriorly were marked with a negative sign. Right: The illustration depicts the ideal HOT in relation to the medial tendon structures.

Osteotomies tangent to the posterior rim of the middle facet (pMf) and the anterior rim of the aspect of the posterior facet (aPf) were simulated with each entry point (Figure 3).

As a result, the range between these osteotomies indicates the direction without affecting the facets and is called the “safe zone.” Angles of all the osteotomies were measured in relation to the reference (Figure 3).

The osteotomy depth was defined as the distance from the lateral wall to the flexor hallucis longus (FHL) muscle tendon, which runs right under the sustentaculum tali.

**Figure 4.**
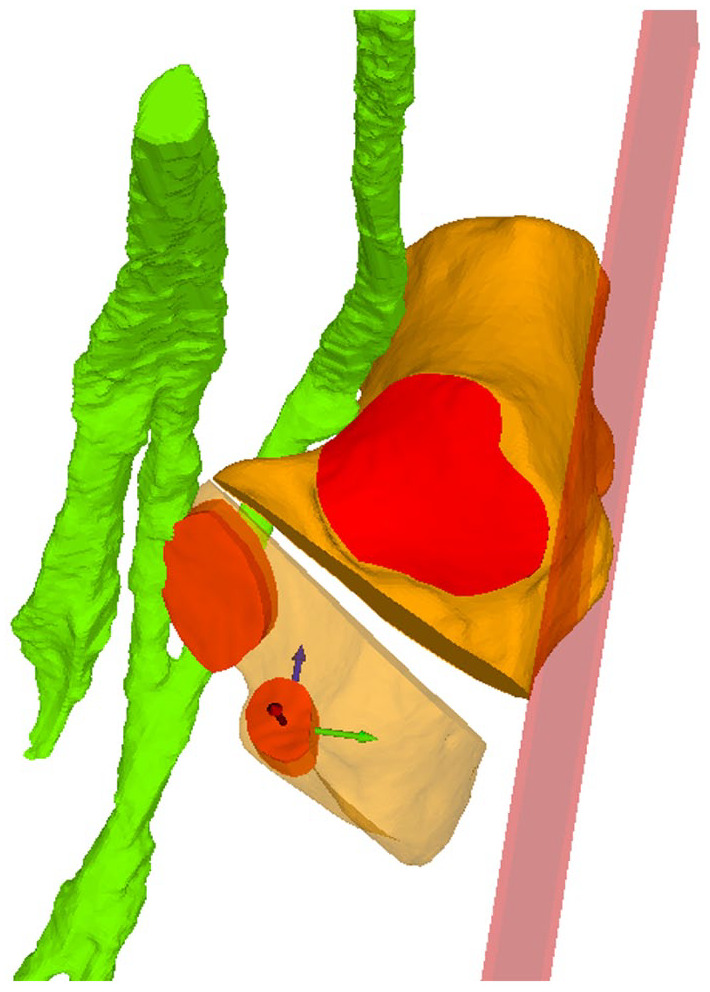
Illustrated is the ideal osteotomy with a 8-mm opening. The anterior facet follows with a standardized coordinate system used to quantify the degree of translation.

The ideal osteotomy, defined as the bisecting angle between the aMf and aPf, was simulated. The fragment to be osteotomized was rotated around an axis located at the medial border of the osteotomy. A standard lengthening of 8 mm was simulated, which is an average correction, and meant a rotation of the fragment of 10 degrees. To quantify the change of the fragment, the translation of the anterior facet was measured (Figure 4). Therefore, an auxiliary coordinate system aligned with the osteotomy plane (X-axis), the lateral calcaneus wall (Z-axis), and its location in the center of the anterior facet was created.

This allows precise calculation of the translation and rotation in each axis and comparing it between osteotomies (Figure 4). Two of the authors (PRF and AK) performed osteotomy simulation and computational analysis.

### Statistics

Because these are metric data, the average of the values from both readers was used.

The Kolmogorov-Smirnov test was used to test the data for a normal distribution. The difference for metric data was calculated using T-testing for normally distributed data or the Friedman test with post hoc testing otherwise. Pearson correlation test was used to identify correlations. Interreader reliability was quantified in terms of the intraclass correlation coefficient (ICC) based on a 2-way random effects model. All the results are given as the mean of both the readers. Significance was set at *P* <.05. Analyses were performed using IBM SPSS Statistics for Apple, version 27.0 (IBM Corporation, Armonk, NY).

## Results

**Figure 5.**
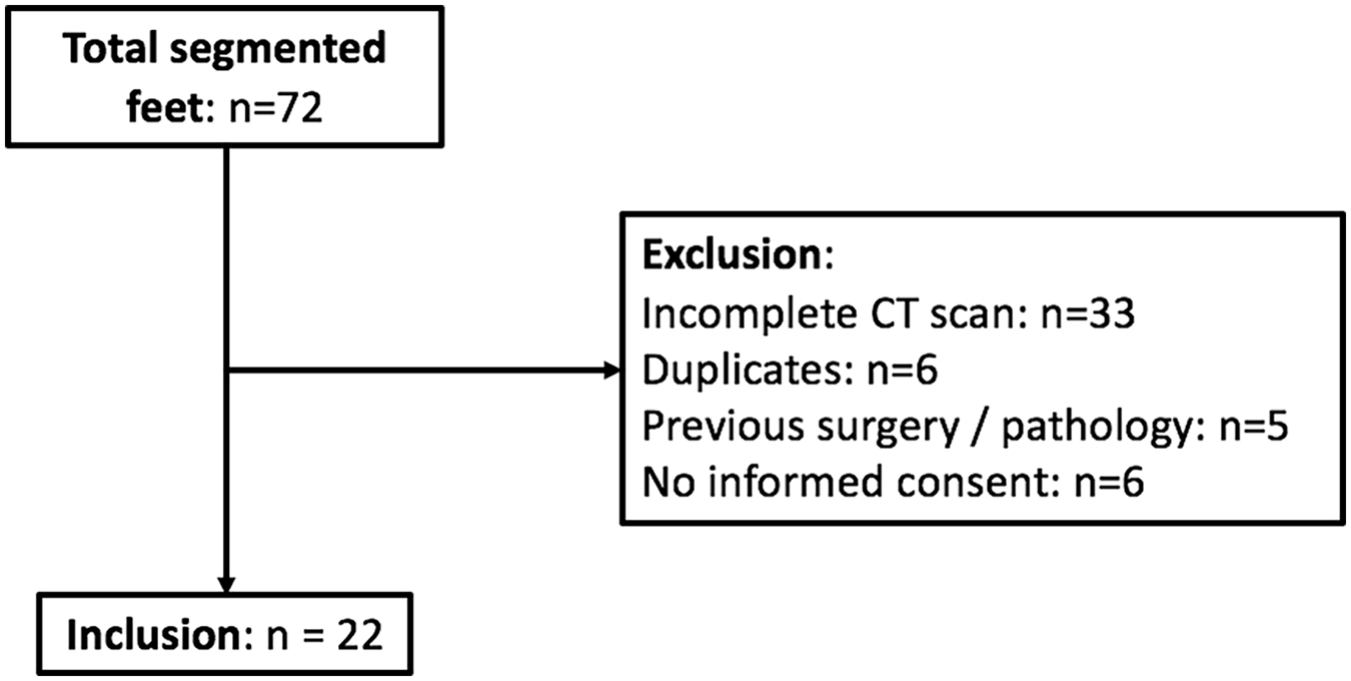
Patient population.

Of the initial 72 patients, 22 were included in our final analysis (Figure 5). The FHL tendon could also be segmented in 19 of these patients; however, technical difficulties limited segmentation in 3 cases. None of the patients had a fused posterior and medial facet. CT scans were taken at a neutral position of the foot (93 ± 9.3 degrees), which corresponds to the intraoperative position in which the foot is held when orienting for the osteotomy

### Calcaneal Osteotomy Angle

**Figure 6.**
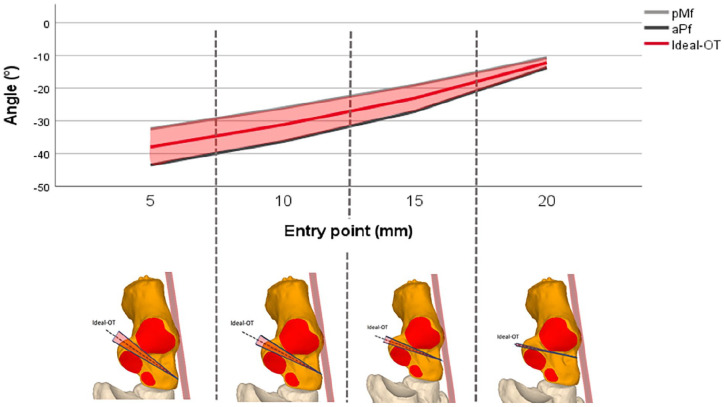
The figure shows the ideal osteotomy and the safe zone (red-highlighted) depending on the different entry points.

The osteotomy results are presented in Table 1 and Figure 6. If the entry point is 20 mm behind the CC joint, osteotomy without facet penetration was impossible in 23% of cases.

**Table 1.** Osteotomy Results.^a^

x	20 mm	15 mm	10 mm	5 mm
pMf (degrees)	−10.7 (−17.8 to 2.5 ± 5.5)	−19.1 (−27.5 to 3.5 ± 10.6)	−26.0 (−34.5 to −14.5 ± 4.4)	−32.4 (−41.0 to −22.0 ± 6.5)
ICC	0.652	0.933	0.952	0.949
aPf (degrees)	−13.7 (−27.5 to 3.5 ± 10.0)	−27.1 (−37.5 to −13.0 ± 6.5)	−36.3 (−46.5 to −24.5 ± 6.5)	−43.4 (−53.0 to −31.5 ± 6.5)
ICC	0.835	0.932	0.925	0.918
Ideal osteotomy (degrees)	−12.3 (−21.5 to 1.75 ± 7.4)	−23.1 (−32.0 to −10.8 ± 5.4)	−31.2 (−40.5 to −18.5 ± 4.9)	−37.9 (−47.0 to −26.8 ± 4.6)
Range of safe zone (degrees)	3.0 (16.0 to 10.0 ± 6.5)	8.0 (16.0 to 2.0 ± 4.0)	10.2 (16.0 to 4.0 ± 10.2)	11.0 (15.0 to 6.5 ± 2.6)
Intraarticular Hintermann osteotomies, n (%)	5 (22.7)	0 (0)	0 (0)	0 (0)

Abbreviations: aPf, anterior edge of the posterior facet; pMf, posterior edge of the middle facet.

a Data are reported as mean (min-max ± SD) unless otherwise indicated. The measured parameters as a function of the various entry points. The angles are positive if the osteotomy was anterior to the 0 degrees reference line, and negative if it was posterior to the reference osteotomy, as illustrated in Figure 3.

The ideal osteotomy shows significant differences depending on the entry point (*P* ≤ .01). There are also significant differences between each safe zone of the different entry points (*P* ≤ .01). There was no correlation found between the angles to be osteotomized, nor the size of the safe zone and the length of the patients’ calcanei.

### Distance to the Flexor Hallucis Longus Tendon

With an entry point of 20 mm behind the CC joint, the average distance from the lateral wall to the first tendinous structure was 35.4 mm along the pMf and 36 mm along the aPf (Table 2).

**Table 2.** Average Distance in Millimeters From the Lateral Wall to the FHL Tendon Penetration for the HOT.

x	20 mm	15 mm	10 mm	5 mm
pMf bis FHL (mm)	35 (29-59 ± 6.3)	37 (31-60 ± 6.4)	39 (33-57 ± 5.3)	42 (35-65 ± 6.5)
aPf bis FHL (mm)	36 (29-59 ± 6.4)	38 (31-59 ± 6.1)	42 (34-56 ± 5.0)	46 (39-65 ± 5.9)

Abbreviations: aPf, anterior aspect of the posterior facet; HOT, Hintermann osteotomy; pMf, posterior aspect of the middle facet.

a Data are reported as mean (min-max ± SD) unless otherwise indicated.

In the patient with the shortest distance, it was 29 mm to the FHL. If the HOT is performed at an entry point of 5 mm behind the CC joint, the distance reaches 41.5 mm at the pMf and 46.1 mm at the aPf. Different entry points lead to statistically significant differences (*P* ≤ .05) between all the osteotomy depths, except between aPf 20 mm and aPf 15 mm (*P* = .059).

### Translation of the Anterior Facet

**Figure 7.**
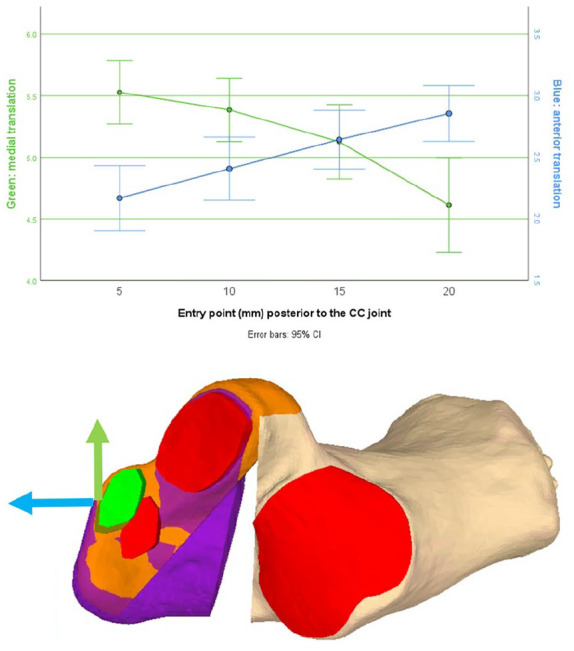
Top: Illustration of the anterior facet transformation as a function of the HOT’s entry point. Blue represents anteroposterior translation, indicating that the elongation of the lateral column increases with distance from the CC joint. The mediolateral alignment is represented by the Green dots and connecting line. The medialization reduces with increasing distance from the CC joint. Bottom: An osteotomy with a 20-mm entry point and one with a 5-mm entry point are shown. The anterior facets and translocated calcanei are seen, red the original facet before the osteotomy, green after the osteotomy. The green and blue arrows represent the translation seen in the top graph. HOT, Hintermann osteotomy.

The anterior facet undergoes distal translation to lengthen the foot’s lateral column, as well as medialization, because of incomplete osteotomy and fragment rotation. At the entry points of 20, 15, 10, and 5 mm, the anterior translation was 2.9, 2.6, 2.4, and 2.2 mm, respectively, and the medial translation was 5.5, 5.4, 5.1, and 4.6 mm, respectively (Figure 7). The medialization of the fragment is significant between each entry point (*P* ≤ .03).

## Discussion

The findings of our study highlight the importance of the correct entry point of HOT. Even if the osteotomy angle is precisely calibrated, the HOT with an entry point of 20 mm posterior to the CC joint results inevitably in 23% of cases in iatrogenic articular damage. When considering the point at which the osteotomy is initiated, it seems easier if the HOT is started as close as possible to the CC joint in order to avoid damaging the articular facets. The entry point has a significant impact not only on the osteotomy angle but also on the safe osteotomy depth, as well as on the translocation of the osteotomized fragment.

The literature lacks consensus on the arbitrarily chosen entry point, despite its significant impact.^8,10-13,15,17^ The more anterior the osteotomy, the greater the margin of error for the HOT, with an average of 11 degrees with a 5-mm entry point and 3.3 degrees with a 20-mm entry point. An angle for the direction in which joint penetration never occurs in any of the cases could not be found, indicating a high interindividual difference in morphology. In a recently published study, distance mapping was used to characterize the facet joints and thus the articular surfaces, and it appears to work very well and reliably.^15^ The osteotomy’s simplified measurement in comparison to the lateral wall was calculated using a single, predefined entry point between the facets. They chose 16 mm behind the CC joint. The safe angle along the posterior facet was calculated as 68 degrees using only 1 entry point. The lateral wall is represented as 0 degrees in their analysis, yet in our study it is labeled as 90 degrees. Converting this to our data yields an angle of −22 degrees, which is comparable to the −23 degrees we calculated for a 15-mm entry point.

The clinical relevance of facet penetration has yet to be determined, but the authors of this publication assume it does have an impact. The penetration of the medial facet should not be underestimated and is somewhat overlooked, as it absorbs large load-bearing forces over a very small area.^18^

The distance to the important neurovascular structures at risk on the medial side of the calcaneus varies depending on the entry point. In our study, the safe osteotomy distance was defined as the distance to the closest medial structure at risk, which in our case was 100% of the FHL tendon, with the understanding of other structures on the medial side, such as the medial plantar branch of the nerve, which was not considered in our case.^17^ However, this neuronal structure was never injured in prior examinations in the case of the Evans osteotomies.^17^ In the individual cases where we were also able to segment the branch of the posterior tibial artery, it was clearly further away than the FHL tendon. In our simulation, the FHL tendon was thus exposed to the greatest danger, as described in previous studies.^12^ The distance to the FHL extends as the entry point moves further anterior. With an entry point 20 mm posterior to the CC joint, the osteotomy saw blade can make contact with the FHL after 29 mm and after 35 mm at 5 mm behind the CC joint in the shortest case. Our novel calculating method could help to reliably determine the safe osteotomy depth preoperatively, perhaps preventing tendon penetration.

The extension of the lateral column alters the foot’s biomechanics, which is influenced by the entry point and direction of the osteotomy, the size and direction of the osteotomy, and the shape of the graft.^19-23^ In our study, we were able to demonstrate the differences between the various osteotomy directions through translation. A clinical study demonstrated that the Evans osteotomy causes more degeneration in the CC joint than the HOT.^6^ We discovered that a posterior entry point resulted in a greater anterior displacement in the anterior calcaneus (eg, 2.9 mm for a 20-mm entry point vs 2.2 mm for a 5-mm entry point). Although our study cannot provide direct implications for CC joint stress, a greater anterior displacement of the anterior fragment would clearly increase CC joint strain. This is in contrast with earlier biomechanical research. In a newly published finite element research, a reduced load was reported, and they infer that the HOT leads to increased peak pressure in the CC and talocalcaneal joints compared with the Evans osteotomy.^22^ However, our results support the clinical findings that less degeneration in the CC joint was found in HOT compared with Evans osteotomies.

Other biomechanical studies found that lateral column lengthening reduced peak pressures in the CC joint, which does not correspond to the other findings.^23^ Compared with the HOT, the Evans osteotomy also improved the contact area and forces in the posterior subtalar facet.^22^ However, this publication also demonstrated that the increased load in the CC joint favored the reduction of load on the talonavicular joint. The impact of our findings on biomechanics is unclear and requires further clarification.

Our study has limitations. The scans, and therefore our analysis, were performed on nonweightbearing, supine patients. This certainly influenced the osteotomy’s direction, but not the osseous structures. The CTs were taken at 93 degrees, neutral position, which is similar to how the foot is held intraoperatively when referencing for the HOT osteotomy. All were nonweightbearing CTs, as intraoperatively there is no weightbearing as well. Further, we performed an osseous analysis that ignored any ligamentous structures. This, in our opinion, has little influence on the anatomical analysis of the facet joints, but the ligaments do have a significant impact on the osteotomy and translation. Especially subtalar interosseous ligaments are important and are at highest risk when the osteotomy is performed.^24^ The osteotomy was also calculated with a 5-mm entry point, although this is a rather theoretical calculation because fixing the anterior fragment with such an osteotomy may be challenging. However, this helps to clearly demonstrate the problem and extrapolates the data. Although our study shows that the fragment is translated very accurately and reproducibly, it remains to be seen what happens to the rest of the foot during such a HOT and how this affects its biomechanical properties in dependence on the entry point. The calculation was carried out on healthy feet, which were used as a template for osteotomies, typically after fracture malunion of the contralateral side. No specific planovalgus feet were investigated. Finally, no angle has been defined at which facet penetration will never occur in any of the cases. This supports the need of individual patient-specific operation planning, which may then be guided by a personalized operation system, which is currently unavailable.

## Conclusion

We conclude that beginning the HOT within 15 mm of the calcaneocuboid joint minimizes facet-penetration risk, widens the intraoperative forgiveness angle, and increases clearance from medial neurovascular structures. Posterior starts (≥20 mm) should be avoided or planned with patient-specific navigation.

## Supplemental Material

sj-pdf-1-fao-10.1177_24730114251363916 – Supplemental material for The Importance of the Entry Point and Osteotomy Direction in Calcaneal Lengthening Osteotomy Supplemental material, sj-pdf-1-fao-10.1177_24730114251363916 for The Importance of the Entry Point and Osteotomy Direction in Calcaneal Lengthening Osteotomy by Pascal Raffael Furrer, Arnaud Klopfenstein, Silvan Beeler, Arnd Fredrik Viehöfer and Stephan Hermann Wirth in Foot & Ankle Orthopaedics
